# Supplementary material for: Optimization of a Sustainable Protocol for the Extraction of Anthocyanins as Textile Dyes from Plant Materials
Source: Molecules. 2021 Nov 9;26(22):6775. doi: 10.3390/molecules26226775 (PMC8625177; doi:10.3390/molecules26226775)
Supplement: Supplementary file 1 [file molecules-26-06775-s001.zip › molecules-1423862-supplementary.pdf]

molecules

MDPI

Supplementary Material

Optimization of a Sustainable Protocol for the Extraction of Anthocyanins as Textile Dyes from Plant Materials

Elisa Gecchele [1], Stefano Negri [1], Anna Cauzzi [1], Anna Cuccurullo [1], Mauro Commisso [1], Alessia Patrucco [2], Anastasia Anceschi [2], Giorgio Zaffani [3] and Linda Avesani [1,*]

[1] Department of Biotechnology, University of Verona, Strada Le Grazie 15, 37134 Verona, Italy; elisa.gecchele@univr.it (E.G.); stefano.negri@univr.it (S.N.); anna.cauzzi@hotmail.com (A.C.); anna.cuccurullo@univr.it (A.C.); mauro.commisso@univr.it (M.C.)

[2] CNR-STIIMA, Italian National Research Council, Institute of Intelligent Industrial Technologies and Systems for Advanced Manufacturing, Corso G. Pella 16, 13900 Biella, Italy; alessia.patrucco@stiima.cnr.it (A.P.); anastasia.anceschi@stiima.cnr.it (A.A.)

[3] Cooperativa Sociale Cercate, Via Bramante 15, 37134 Verona, Italy; g.zaffani@ecofarm.storti.com

* Correspondence: linda.avesani@univr.it; Tel.: +39-0458027839

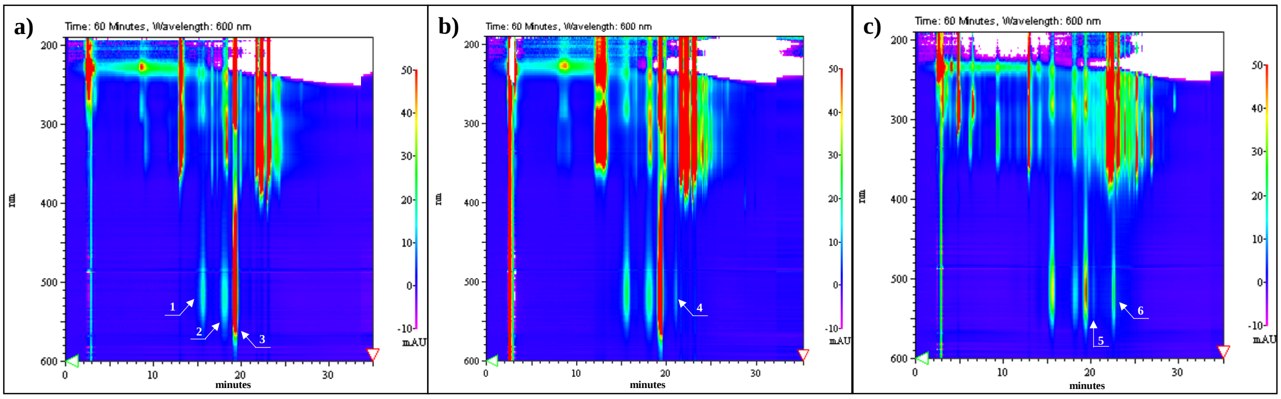

Figure S1. Anthocyanin profile obtained by HPLC-DAD data at 520 nm. a) 3D chromatogram of a red chicory ethanol extract (12.5%, v/v) showing the three main anthocyanins. b) methanol red chicory extract showing the fourth anthocyanin. c) 3D chromatogram of a lyophilized red chicory extract, stored for 6 months at 23°C and solubilized with acidified water.
